# Regular Meeting Fulton County Medical Society, June 17, 1915

**Published:** 1915-06

**Authors:** Allen H. Bunce


### REGULAR MEETING FULTON COUNTY MEDICAL SOCIETY, JUNE 17th, 1915.

By Allen H. Bunce, M. D.

Dr. James N. Brawner read a paper on the "Alcoholic Psychoses." It appears elsewhere in the Journal. Dr. Hansell Crenshaw said that chronic alcoholism when uncomplicated by other diseases is easier to treat successfully than most physicians believe and that the sot is not always hopeless. Dr. Crenshaw said he believed that practitioners generally would be agreeably surprised to observe at first hand the prompt improvement and recovery of most sufferers from alcoholic psychoses as managed and treated in any properly equipped institution. The question of whether cured patients relapse or not after leaving the hospital is a problem dependent upon psychic and sociologic factors; because the same worry, over work or domestic infelicity which caused the original addiction to alcohol may again become operative and cause the patient to relapse despite the fact that his craving for alcohol has been absolutely abolished. Dr. Crenshaw emphasized the importance of looking for other diseasess besides addiction to alcohol in treating these psychoses, because the alcoholic psychoses are seldom uncomplicated by other diseases and are more frequently the effect than the cause of other pathological conditions. Syphilis so often complicates chronic alcoholism that a Wasserman should be done on the blood and spinal fluid of every case that shows mental symtoms.

Dr. L. M. Gaines said alcohol is the most remarkable fluid in the world. It occurs universally and in many different forms. Even among savage tribes its use is not unknown. Its use and abuse creates political, medical and social questions. It affects all tissues of the body—the kidney, liver, stomach, and especially the brain. An alcoholic psychosis is produced by an extraordinary effect of alcohol on the brain. Dr. Gaines thinks alcoholic epilepsy should be considered in this connection. He reported two recent cases of alcoholic epilepsy where other conditions were absolutely excluded. Another very important point to be kept in mind is the effect of trauma on the brain when it is surcharged with alcohol. Not infrequently alcohol plus trauma equals insanity. Dr. Gaines cited several cases illustrating this effect. Finally he advocated a careful study and thorough examination of each individual case of alcoholism so as to be better able to benefit each individual. .

Dr. M. T. Davis said alcohol may beget ills other than drunkenness. Epilepsy, insanity, chronic neuralgia, and many other diseases may be caused either directly or indirectly by alcohol. Heredity is a very important factor to be considered. The offsprings of alcoholics may have imperfectly organized brains whereby the moral perceptions are hindered, in common with the intellectual capacity. Many of these may never take a drink of alcohol but still be inebriates and ofttimes criminals. The offsprings of alcoholics should be studied carefully. These children should receive special care and training early in life so that they may become well developed morally, physicallly and mentally. By doing this we may so strengthen them that they will become strong and useful citizens.

Dr. Duncan said he had had most remarkable results with the Tr. of Gelsemium in the treatment of alcoholics. He also advocated Tr. Digitalis in large doses, even as high as 60 drops in some instances. With these he often combines Chloral. He gave an example of a shoemaker with delirium tremens who was given 60 grains Chloral, 60 drops digitalis together with the Tr. of Gelsemium with the remarkable result that the patient was able to go to his work the next day.

Dr. J. C. McDougall read a paper on "Empyema of the accessory sinuses.

It appears elsewhere in the Journal.

Dr. Lokey said that the paper of Dr. McDougall was very practical and stressed several very important points. Empyema of the accessory sinuses is freqeuntly overlooked especially in persistent headaches and neuralgia. He has had an unusually large number of cases affecting the maxillary sinus this year which he thinks is due to the epidemic of influenza which occurred here last winter. In cultures which Dr. Lokey has had made from his cases he has found the influenza bacillus, the staphylococcus and the streptococcus as the chief offending organisms. He reported one case where the colon bacillus was found and which was very resistant to treatment. Finally he succeeded in curing this case with a solution of permanganate of potash. Acute cases of empyema are treated by drainage and irrigation. In the chronic cases it is necessary to do a more radical operation.

Dr. Howard Hall said these cases of empyema of the accessory sinuses usually begin with a slight rhinitis and it is only the neglected cases which develop an empyema. He emphasized the fact that the various sinuses form a vicious circle in that infection is carried from one to another, etc. He considers that the infection usually starts in the anterior ethmoidal cells. The ethmoidal drains into the maxillary, therefore we see so many infected maxillary sinuses. Finally, Dr. Hall said the anterior ethmoidal cells should always be investigated first to see whether or not they furnish the focus of infection.

Dr. John Funke discussed the bacteriology of infection of the accessory sinuses. He said that the organisms which usually produce the pus found are the staphylococcus, pneumococcus and the streptococcus. While the influenza bacillus may be the direct cause of the trouble, it does not produce any pus itself, but furnishes a favorable field for the development of the pyogenic organisms.

Dr. Clarance A. Rhodes made "A Preliminary Report on the Examination of Mother's Milk in some Nutritional Disturbances in Infants."

---

It is not reasonable to believe that a retroposed uterus by its mere weight upon the bowel can cause constipation.—American Journal of Surgery.

---

Simple retroposition of the uterus is quite normal to many women and should not be "corrected" without clear indications.—American Journal of Surgery.

---

Patients suffering with sciatica should be examined carefully for a possible lesion of the pelvic bones, the vertebrae or the spinal cord iteslf.—American Journal of Surgery.

---

Among the diagnostic possibilities of a flattened or rounded mass in the midline of the abdomen it is worth remembering fused or horeshoe kidney. The diagnosis can be established by pyelography.—Aemrican Journal of Surgery.

---

The occurrence of dermoids in the midline of the perineum is too often forgotten. Situated at the anterior anal margin a dermoid cyst be mistaken for a hemorrhoid, or if discharging through a sinus, for an ordinary "fistula in ano." Situated more anteriorly the sinus is often mistaken for a urethral fistula.—American Journal of Surgery.

